# Effect of Compound Chuanxiong Capsule on Inflammatory Reaction and PI3K/Akt/NF-*κ*B Signaling Pathway in Atherosclerosis

**DOI:** 10.1155/2015/584596

**Published:** 2015-10-11

**Authors:** Qunfu Kang, Weihong Liu, Hongxu Liu, Mingxue Zhou

**Affiliations:** ^1^Beijing Hospital of Traditional Chinese Medicine, Capital Medical University, Beijing 100010, China; ^2^Beijing Institute of Traditional Chinese Medicine, Beijing 100010, China

## Abstract

Compound Chuanxiong Capsule (CCC), a Chinese herbal compound, can exhibit antiatherosclerotic effect; however, its mechanism is still unclear. This study is designed to study the mechanism of CCC on atherosclerosis in the ApoE-knockout (ApoE^−/−^) mice fed with a high-fat diet. After 6 weeks of high-fat feeding, 40 ApoE^−/−^ mice were randomized (*n* = 10) and treated with lipitor, high-dose or low-dose CCC, or distilled water (ApoE^−/−^ group) for 7 weeks. The blood lipids in serum and the plaque areas of the mice were measured and the mRNA expressions of phosphatidylinositol-3-kinases (PI3K), Akt, nuclear factor-kappa B (NF-*κ*B), tumor necrosis factor-*α* (TNF-*α*), and interleukin-6 (IL-6) of the aortae were determined. The data showed that CCC can significantly decrease the levels of blood lipids, atherosclerosis index, and plaque areas and increase collagen proportion in plaques as compared with the untreated mice (*p* < 0.05, *p* < 0.01). In addition, CCC can significantly reduce the mRNA expressions of PI3K, Akt, NF-*κ*B, IL-6, and TNF-*α* in the mice fed with a high-fat diet (*p* < 0.001). Thus, we concluded that CCC can inhibit inflammatory reaction in the ApoE^−/−^ mice fed with a high-fat diet. This mechanism may be attributed to regulating PI3K/Akt/NF-*κ*B signaling pathway.

## 1. Introduction

Atherosclerosis, a chronic inflammatory disease, plays a key role in the occurrence and development of the cardiovascular diseases, which has become as the most chronic cause of death worldwide [1]. The data from the report of Cardiovascular Disease in China 2013 showed that cardiovascular disease resulted in 41.1% deaths in the city and 38.7% in the rural areas [2]. Therefore, it is essential to prevent and treat atherosclerosis for the better prognosis of the patients with cardiovascular diseases.

In the inflammatory mechanism of initiating the occurrence of atherosclerosis, NF-*κ*B plays one of the most important roles as multifunctional transcription regulators. Once activated, it exerts crucial effects on inflammatory reaction, immune reaction, cell proliferation, and apoptosis by regulating gene expressions of inflammatory cytokines and chemokines, such as IL-6 and TNF-*α* [3]. The expressions of NF-*κ*B and its cascade genes can be regulated by PI3K/Akt signaling pathway, which plays a vital role in the process of cell proliferation, apoptosis, and inflammatory reaction [4, 5].

CCC, one of the typical Chinese herbal compounds with the potential of activating blood circulation, is composed of Chuanxiong (*Ligusticum*) and Danggui (*Angelica sinensis*). Zhao and Niu showed that CCC can decrease the level of blood lipids in the patients with coronary heart disease [6] and can reduce the risk of heart failure, for example, the frequency and severity, and improve clinical symptoms of cardiac insufficiency [7]. In rat atherosclerosis model, it was found that CCC could increase the coronary blood flow, inhibit platelet adhesion and aggregation, reduce blood viscosity, and improve the state of blood stasis in blood vessels [8].

The mechanism of action of CCC, in TCM, is attributed to the potential of Chuanxiong and Danggui in activating blood circulation. Chuanxiong and Danggui have been widely used in the diseases with blood stasis syndrome [9]. It was reported that CCC can inhibit the aggregation of platelets and lower the viscosity of the blood [10]. However, the mechanism of action of CCC on atherosclerosis is still unclear.

Considering the role of PI3K/Akt/NF-*κ*B signaling pathway and its downstream inflammatory cytokines in atherosclerosis, this study was designed to investigate the inflammatory mechanism of CCC on atherosclerosis in the ApoE-knockout (ApoE^−/−^) mice fed with a high-fat diet by detecting the expressions of TNF-*α*, IL-6, PI3K, Akt, and NF-*κ*B.

## 2. Materials and Methods

### 2.1. Animals and Treatment

Eight-week-old male ApoE^−/−^ mice (*n* = 40, 18–20 g) on a C57BL/6J background and 10 C57BL mice introduced and bred by the Animal Unit of Peking University Health Science Center were used in the study. The housing and care of the animals and all the procedures were performed in accordance with the guidelines and regulations of the University of Bristol and the United Kingdom Home Office.

### 2.2. Ethics Statement

This study was approved by the Institutional Animal Care and Use Committee (IACUC) from Peking University Health Science Center.

### 2.3. Materials and Reagents

A TRIzol kit was purchased from Invitrogen Company (California, USA), PCR primers were synthesized by Sangon Biotech Co., Ltd. (Shanghai, China), and an M-MLV RT kit and a real-time (RT)-PCR kit were purchased from Takara Company (Otsu, Shiga, Japan). The blood lipid kits were purchased from Zhongsheng Beikong Biotechnology Co., Ltd. (Beijing, China) to measure total cholesterol (TC), triglycerides (TGs), low-density lipoprotein (LDL), and high-density lipoprotein (HDL-C). Van Gieson (VG) staining kit was purchased from MAIXIN-BIO (Fuzhou, China). CCC was purchased from Shandong Phoenix Pharmaceutical Co., Ltd., (Shandong, China; Batch number: Z20000035) and lipitor (atorvastatin) was purchased from Pfizer Pharmaceutical Co., Ltd. (Dalian, China; Batch number: H20051408).

### 2.4. Establishment of Atherosclerosis Model

All the ApoE^−/−^ mice were fed with a high-fat diet containing 21% (wt/wt) fat from lard supplemented with 0.15% (wt/wt) cholesterol [11] and obtained from Beijing Ke'ao Xieli Feed Co. Ltd. (Beijing, China) for 13 weeks. Additionally, 10 C57BL mice fed a standard chow diet containing 4% fat were used as normal control group. All the mice were inspected at least once every 24 h.

### 2.5. Drug Treatment

After 6 weeks of high-fat diet, the ApoE^−/−^ mice were randomized into ApoE^−/−^ (*n* = 10), ApoE^−/−^ + lipotor (*n* = 10), ApoE^−/−^ + CCC low (*n* = 10), and ApoE^−/−^ + CCC high (*n* = 10) and were treated with 2.973 mg/kg/d lipitor (positive-control drug) by intragastric administration, high dose of CCC (1333.48 mg/kg per day), low dose of CCC (333.37 mg/kg per day), or distilled water (control group) for an additional 7 weeks. The feeding of the mice in all the groups was accompanied by a high-fat diet. The choice of medical doses was based on the clinically relevant doses in humans (the conversion coefficient between human and mice is 9.01, and the medical doses in mice are 9.01 × the clinically medical doses in humans [12]). Distilled water was used to dilute the medicine. Distilled water consumption was monitored twice weekly, and drug concentration was adjusted as required.

### 2.6. Histology

All the mice in the five groups were euthanized with 0.1% pentobarbital sodium. The heart from each mouse was removed and the one-third of the apical heart, including the aortic sinus, was fixed in 10% formaldehyde, embedded, and sectioned to determine the morphology of any atherosclerotic plaque by hematoxylin and eosin (HE) and VG staining.

### 2.7. Evaluation of Atherosclerotic Lesions

To quantitatively evaluate atherosclerotic lesions, eight sections (5 *μ*m-thick) per group were selected and the sections in the same segment were quantified according to Suzuki et al. [13]. Ten slides per group were examined for morphometric analysis. The average of the measured four sections per sample was recorded. The plaque morphology and the collagen content of atherosclerotic plaque were evaluated by HE and VG staining. A morphometric analysis was performed using Image Pro Plus (Media Cybernetics, Rockville, MD, USA). The plaque area was measured directly and was subtracted from the area enclosed by the internal elastic lamina to derive the patent lumen area [14], but the plaque area should be corrected by dividing the internal elastic lamina surrounding area.

### 2.8. Determination of Plasma Lipid Concentration

Blood samples were drawn from the left ventricle of a cohort of all male ApoE^−/−^ mice that received a high-fat diet for 13 weeks. TC and TGs were determined by enzyme studies in serum. LDL-C and HDL-C levels were determined by immunoturbidimetry. Finally, all the indices were determined using the RX-2000 radiometer (Technicon Instruments Company, Tarrytown, NY, USA), and the atherosclerosis index (AI) was calculated using the formula [15]:

(1) $$\begin{array}{c} AI=\frac{non\text{-}HDL\text{-}C}{HDL\text{-}C}. \end{array}$$

### 2.9. Real-Time Quantitative PCR

The aortic root from each mouse from all the groups was removed and stored in −80°C to examine the mRNA expressions of PI3K, Akt, NF-*κ*B, IL-6, and TNF-*α*. The total RNA from aortae was extracted using TRIzol kit according to the manufacturer's instructions. The primers of PI3K, Akt, NF-*κ*B, IL-6, and TNF-*α* are shown in Table 1. The protocol used for RT-PCR was similar to the previously described method [16]. The model of qPCR machine was ABI 7500 (America) and the program used was SDS 1.3 version. The data was analyzed by 2^−ΔΔCT^ method, and the internal reference gene used was glyceraldehyde 3-phosphate dehydrogenase (GAPDH).

### 2.10. Statistical Analysis

Mean values and standard deviations (mean ± S.D.) were calculated for each of the variables. All the statistical procedures were performed using Statistical Package for the Social Sciences (SPSS) 11.5. Normally distributed data was analyzed using one-way analysis of variance (ANOVA), while the statistical significance of intergroup differences in all the tested variables was evaluated using Bonferroni post hoc test. In all the cases, *p* < 0.05 was as statistically significant.

## 3. Results

### 3.1. High-Fat Diet Induced Atherosclerosis in ApoE^−/−^ Mice Model

**Figure 1:**
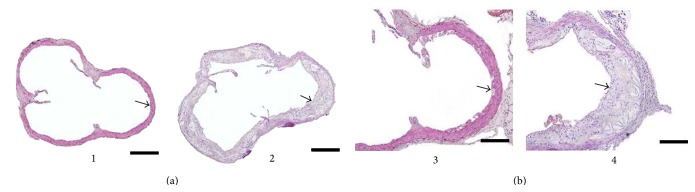
Comparison of pathological morphology of the aorta of the C57BL/6J mice and the ApoE^−/−^ mice under different magnification at 13 weeks after being fed with different diets. (a) The pathological morphology of the aortae of the C57BL/6J and ApoE^−/−^ mice under ×100 magnification at 13 weeks after being fed with different kinds of diets (scale bars = 500 *μ*m). (b) The pathological morphology of the aortae of the C57BL/6J and ApoE^−/−^ mice under ×200 magnification at 13 weeks after being fed with different kinds of diets (scale bars = 200 *μ*m). Hematoxylin and eosin (HE) staining: the black arrow indicates the aorta of the mice.

After the ApoE^−/−^ mice were fed with high-fat diet for 13 weeks, the atherosclerotic plaques in the aortic valves attachment sites, including cholesterol crystal and foam cells, were observed in the aortic roots of the ApoE^−/−^ mice, whereas no plaques were observed in the aortic roots of the C57 mice (Figure 1).

### 3.2. Effects of CCC on Blood Lipids

**Figure 2:**
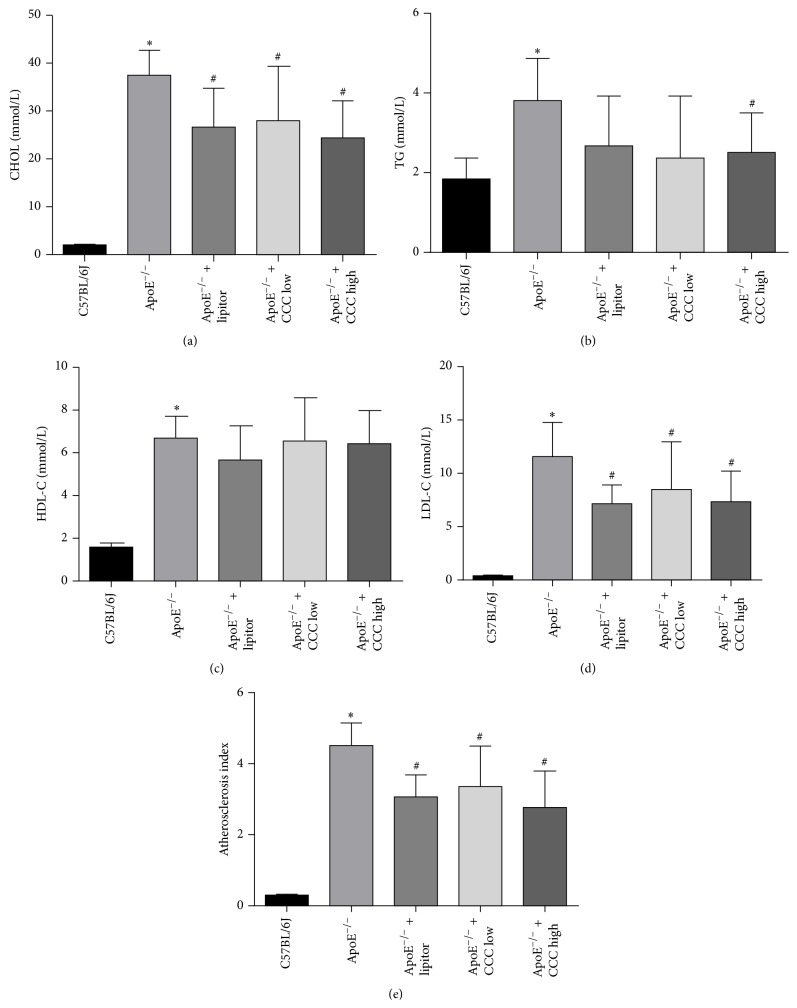
Effects of Compound Chuanxiong Capsule (CCC) on blood lipids in serum of ApoE^−/−^ mice fed with high-fat diet. (a) Total cholesterol (CHOL). (b) Triglyceride (TG). (c) Low-density lipoprotein cholesterol (LDL-C). (d) High-density lipoprotein cholesterol (HDL-C). (e) Atherosclerotic index (AI, AI = non-HDL-C/HDL). ^*∗*^ *p* < 0.05 versus C57BL/6J group; ^#^ *p* < 0.05 versus ApoE^−/−^ group (C57BL/6J: *n* = 10; ApoE^−/−^: *n* = 10; ApoE^−/−^ + lipitor: *n* = 10; ApoE^−/−^ + CCC low: *n* = 10; ApoE^−/−^ + CCC high: *n* = 10).

The results showed that, in comparison with the ApoE^−/−^ group mice, the levels of TGs, TC, and LDL-C in serum of the ApoE^−/−^ mice in the high-dose and low-dose CCC groups were significantly decreased (*p* < 0.05), but the level of HDL-C in serum was not significantly altered (*p* > 0.05) (Figures 2(a)–2(d)).

### 3.3. Effects of CCC on Atherosclerotic Index

The atherosclerotic indexes of mice in the ApoE^−/−^ + lipitor group and the ApoE^−/−^ + CCC low and ApoE^−/−^ + CCC high groups were significantly decreased as compared with the ApoE^−/−^ group mice (*p* < 0.05) (Figure 2(e)).

### 3.4. Effects of CCC on the Atherosclerotic Plaques and Its Compositions

**Figure 3:**
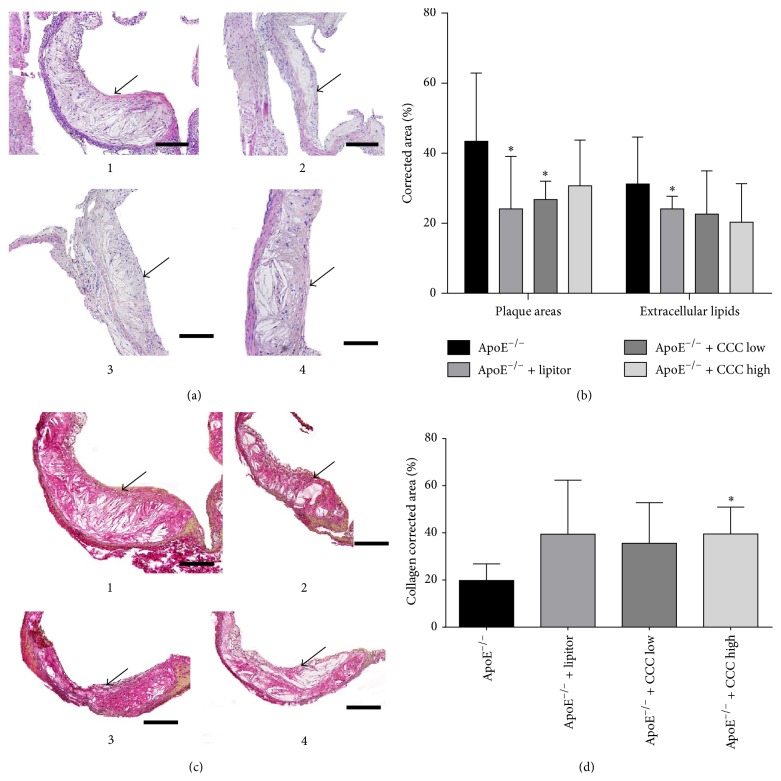
Effect of Compound Chuanxiong Capsule (CCC) on atherosclerotic plaque. (a) Hematoxylin and eosin (HE) staining showing the pathological morphology change of the atherosclerotic plaque in aorta of the ApoE^−/−^ mice after the treatment of CCC. 1: ApoE^−/−^ group; 2: ApoE^−/−^ + lipitor group; 3: ApoE^−/−^ + CCC low group; 4: ApoE^−/−^ + CCC high group. Scale bars = 200 *μ*m, and the black arrow indicates the atherosclerotic plaque in aorta. (b) The statistical analysis of the corrected area of atherosclerotic plaque and extracellular lipids content in plaque of the ApoE^−/−^ mice after the treatment of CCC. ^*∗*^ *p* < 0.01 versus ApoE^−/−^ group. (c) Van Gieson (VG) staining showing the pathological morphology change of content in atherosclerotic plaque in aorta of the ApoE^−/−^ mice after the treatment of CCC. 1: ApoE^−/−^ group; 2: ApoE^−/−^ + lipitor group; 3: ApoE^−/−^ + CCC low group; 4: ApoE^−/−^ + CCC high group. Scale bars = 200 *μ*m, and the black arrow indicates the collagen content in plaque. (d) The statistical analysis of the corrected area of collagen in atherosclerotic plaque of the ApoE^−/−^ mice after the treatment of CCC. ^*∗*^ *p* < 0.01 versus ApoE^−/−^ group (ApoE^−/−^: *n* = 10; ApoE^−/−^ + lipitor: *n* = 10; ApoE^−/−^ + CCC low: *n* = 10; ApoE^−/−^ + CCC high: *n* = 10).

The corrected areas of atherosclerotic plaque in the mice of the ApoE^−/−^ + lipitor and the ApoE^−/−^ + CCC low groups were significantly decreased (*p* < 0.01). Moreover, the area percentage of collagen in the atherosclerotic plaques of the mice in ApoE^−/−^ + CCC high group was significantly increased (*p* < 0.01) as compared to the mice in ApoE^−/−^ group, while the area percentage of extracellular lipid of the two ApoE^−/−^ + CCC groups was decreased, but not significantly changed (*p* > 0.05) (Figure 3).

### 3.5. Effects of CCC on mRNA Expressions of IL-6 and TNF-*α*

**Figure 4:**
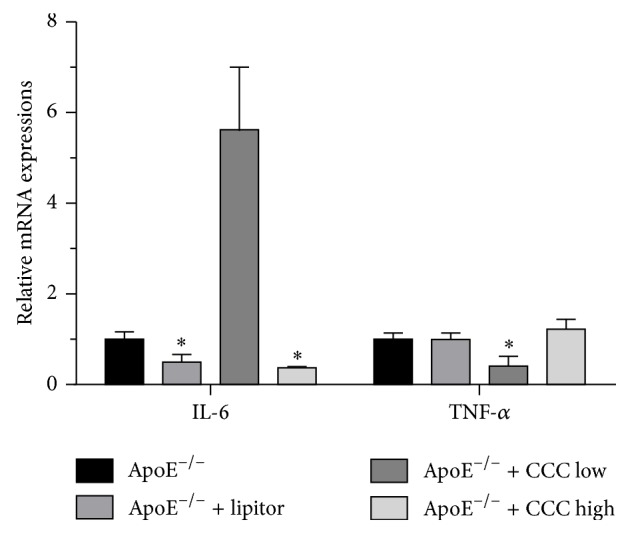
Effect of Compound Chuanxiong Capsule (CCC) on mRNA expressions of IL-6 and TNF-*α* in ApoE^−/−^ mice fed with high-fat diet. ^*∗*^ *p* < 0.001 versus ApoE^−/−^ group. mRNA indicates messenger RNA (ApoE^−/−^: *n* = 10; ApoE^−/−^ + lipitor: *n* = 10; ApoE^−/−^ + CCC low: *n* = 10; ApoE^−/−^ + CCC high: *n* = 10).

As shown in Figure 4, the mRNA expression of IL-6 in mice in the ApoE^−/−^ + CCC high group was more significantly decreased than in mice in the ApoE^−/−^ group (*p* < 0.0001). Additionally, the mRNA expression of TNF-*α* in mice in the ApoE^−/−^ + CCC low group was significantly decreased (*p* < 0.0001).

### 3.6. Effects of CCC on mRNA Expressions of PI3K, Akt, and NF-*κ*B

**Figure 5:**
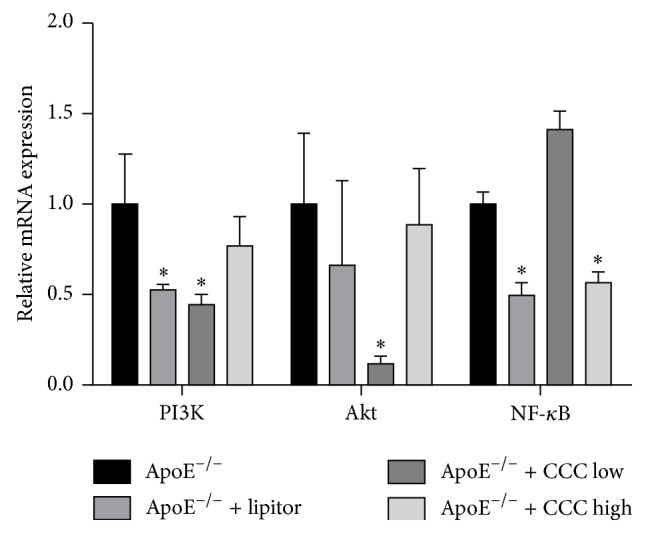
Effect of Compound Chuanxiong Capsule (CCC) on mRNA expressions of PI3K, Akt and NF-*κ*B. ^*∗*^ *p* < 0.001 versus ApoE^−/−^ group. mRNA indicates messenger RNA (ApoE^−/−^: *n* = 10; ApoE^−/−^ + lipitor: *n* = 10; ApoE^−/−^ + CCC low: *n* = 10; ApoE^−/−^ + CCC high: *n* = 10).

As shown in Figure 5, the mRNA expressions of PI3K, Akt, and NF-*κ*B in mice in the ApoE^−/−^ + CCC group were significantly decreased as compared to the mice in the ApoE^−/−^ group (*p* < 0.0001), while the mRNA expressions of PI3K and Akt in mice in the ApoE^−/−^ + CCC low were decreased, but not significantly changed (*p* > 0.05).

### 3.7. Effects of CCC on Protein Expressions of PI3K and p-Akt

**Figure 6:**
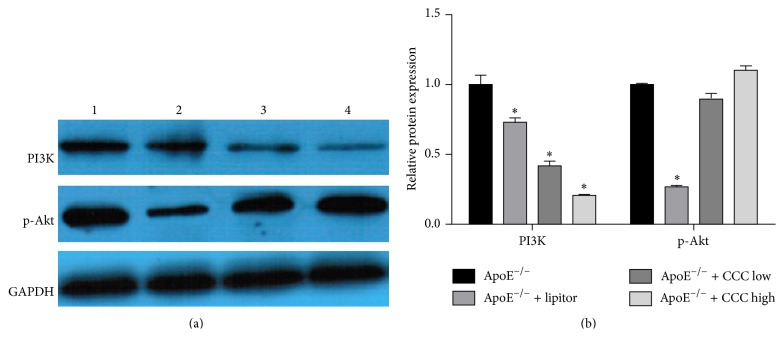
Effect of of Compound Chuanxiong Capsule (CCC) on protein expressions of PI3K and p-Akt. (a) Western blotting (WB) results of PI3K and p-Akt levels in mouse aorta. (b) Quantitative analysis (column diagram) of PI3K and p-Akt levels in mouse aorta based on WB results. ^*∗*^ *p* < 0.05 versus ApoE^−/−^ group (ApoE^−/−^: *n* = 6; ApoE^−/−^ + lipitor: *n* = 6; ApoE^−/−^ + CCC low: *n* = 6; ApoE^−/−^ + CCC high: *n* = 6).

As shown in Figure 6, the protein expression of PI3K in mice in the ApoE^−/−^ + CCC low and high group was significantly decreased as compared to the mice in the ApoE^−/−^ group (*p* < 0.01). The protein expression of p-Akt in the ApoE^−/−^ + CCC low group was decreased, but not significantly changed, as compared to the mice in the ApoE^−/−^ group (*p* > 0.05).

## 4. Discussion

In this study, we have demonstrated that CCC can dramatically ameliorate atherosclerosis in the ApoE^−/−^ mice fed with high-fat diet by improving the blood lipid levels and reducing the plaque areas, which is consistent with the results of the previous studies [7, 10, 17]. In addition, we showed that CCC may inhibit the expression of IL-6 and TNF-*α* by regulating PI3K/Akt/NF-*κ*B signaling pathway in transcriptional level, which may be the main mechanism of CCC exerting the antiatherosclerotic effect.

CCC is one of the typical Chinese herbal compounds, which has been widely used in clinical practices at the physiological relevance dosage of 4T and Tid (666.7 mg/kg per day). It was shown that CCC, at the dosage of 666.7 mg/kg per day, could decrease the blood lipids and C-reactive protein significantly [18] and improves the symptoms of cardiac insufficiency [7, 19]. In this study, the results showed that CCC inhibited atherosclerosis by regulating PI3K/Akt/NF-*κ*B signaling pathway. The potential active ingredients of CCC, we supposed, might be ligustrazine, the main active ingredient of Chuanxiong, and ferulic acid, the main active ingredients of both Chuanxiong and Danggui. Previous studies showed that ligustrazine exhibited protective effects by reducing the levels of TNF-*α* [20] and ox-LDL [21] and others Ferulic acid could significantly decrease TC and the ratio of apo B to apo A and prevent the formation of aortic fatty plaques [22]. However, Xu et al. [23] reported that the contents of ligustrazine in the mixture of Chuanxiong and Danggui were higher than it in the Chuanxiong alone, while Danggui was devoid of ligustrazine. Thus, the inhibitory effect of CCC on atherosclerosis may not be contributed to the individual pharmacological effect of Chuanxiong or Danggui, but the overall effect of CCC, which was consistent with the holistic concept of TCM.

In the present study, the data showed that CCC can significantly decrease the blood lipid, especially TC and LDL-C, in the mice. CCC is composed of Chuanxiong (*Ligusticum*) and Danggui (*Angelica sinensis*); thus its action was attributed to the effects of ligustrazine, the main ingredients of Chuanxiong, or ferulic acid, the main ingredient of both Chuanxiong and Danggui, since the data from the previous studies showed that ligustrazine and ferulic acid can decrease blood lipids and produce a tangible protection in atherosclerosis.

The extracellular lipids and collagen in atherosclerotic plaque were responsible for its stability of atherosclerotic plaque. The data showed that CCC can decrease the percentage of extracellular lipids and increase the percentage of collagen in atherosclerotic plaque. This finding was suggestive that CCC may have the potential effect of promoting the stability of atherosclerotic plaque.

Atherosclerosis has been considered as a chronic inflammatory disease since 1999 [24] and inflammatory factors play an important role in its occurrence and development of atherosclerosis [25–27]. IL-6, an intense inflammatory cytokine, plays a critical role in cardiovascular disease [28], and it can activate endothelial cells and regulate the extracellular lipids [29]. Moreover, IL-6 can induce the synthesis of matrix metalloproteinases and regulate their function to make the atherosclerotic plaques vulnerable [30, 31]. TNF-*α* is a vital cytokine involved in the progress of atherosclerosis [32]. Kivirikko et al. showed that TNF-*α* can inhibit the expression of P4H, which promotes the formation of collagen that makes the atherosclerotic plaques vulnerable [33, 34], and it can inhibit the endothelial cells' function, induce lipid deposition, and promote the generation of inflammatory cytokines to aggravate atherosclerosis [25, 35]. In this study, the data showed that CCC can inhibit the mRNA expression of IL-6 and TNF-*α* in the aorta in the ApoE^−/−^ mice fed with high-fat diet. Based on the effects of IL-6 and TNF-*α* in inflammatory reaction and atherosclerosis, the role of CCC in preventing atherosclerosis may be related to inhibiting the inflammation reaction.

NF-*κ*B is one of the crucial multifunctional transcription regulators. Once activated, it exerts effects on inflammatory reaction, immune reaction, cell proliferation, and apoptosis by regulating the gene expressions of inflammatory cytokines, and chemokines, such as IL-6 and TNF-*α* [3]. Kuang and Wang reported that, in ApoE^−/−^ atherosclerosis mice model fed with high-fat diet, the level of NF-*κ*B was significantly increased and exhibited a positive correlation with inflammation [36], and the results in our study showed that the expression of NF-*κ*B was significantly decreased in the ApoE^−/−^ + lipitor group and ApoE^−/−^ + CCC high group as compared with the model group. In addition, several studies showed that both IL-6 and TNF-*α* were under the influence of NF-*κ*B [37–39] and, in turn, IL-6 and TNF-*α* could promote the activation of NF-*κ*B to result in an inflammatory response [40–42]. In this study, our results showed that CCC can inhibit the expressions of NF-*κ*B in the aorta in mice fed with high-fat diet. These findings suggest that CCC may inhibit inflammatory reaction in atherosclerosis by regulating the expression of NF-*κ*B.

PI3K signaling pathway and its main downstream effector protein kinase B (PKB/Akt) participate in glucose metabolism, differentiation, proliferation, apoptosis, and migration of cells and inflammatory response, and NF-*κ*B is one-key transcription factors in regulating inflammation [43]. PI3K/Akt signaling pathway can enable the NF-*κ*B transcription factors to increase the activity of inflammatory medium gene, which results in the generation of many cytokines. Luo et al. reported that the activation of PI3K/Akt signaling pathway could decrease the levels of reactive oxygen species and lipid deposits that can restrain the formation of plaques to reverse the progress of atherosclerosis [44]. However, it is well known that the activation of PI3K/Akt signaling pathway could induce the aggregation of inflammatory cells to promote the inflammation and accelerate the development of atherosclerosis [45, 46]. Therefore, the inhibitory effect on the PI3K/Akt signaling pathway can inhibit the expression of NF-*κ*B to ameliorate the progress of atherosclerosis [47–49]. Our results showed that both the high-dose and low-dose CCC can inhibit the mRNA expressions of PI3K and Akt, and high-dose CCC can significantly inhibit the mRNA expression of NF-*κ*B in aortae of the ApoE^−/−^ mice fed with high-fat diet. Thus, CCC may suppress the mRNA expression of IL-6 and TNF-*α* by regulating PI3K/Akt/NF-*κ*B signaling pathway in transcriptional level. The results also showed that CCC can significantly inhibit the protein expression of PI3K, but not significantly inhibit the protein expression of p-Akt in aortae of the ApoE^−/−^ mice fed with high-fat diet. The possible explanation is that the dose of CCC did not exert the corresponding effect. Therefore, further investigation is required to explain the possible mechanism of CCC on PI3K/Akt/NF-*κ*B signaling pathway more explicitly.

## 5. Conclusion

The results from the study showed that CCC can dramatically ameliorate atherosclerosis in the ApoE^−/−^ mice fed with high-fat diet. The possible mechanism was attributed to the inhibition of the expression of IL-6 and TNF-*α* by regulating PI3K/Akt/NF-*κ*B signaling pathway. Therefore, it is necessary to further investigate the potential effect and mechanism of CCC preventing the occurrence of atherosclerosis.

## Figures and Tables

**Table 1:** 

Genes	Forward	Reverse
GAPDH	5′-GCAAGTTCAACGGCACAG-3′	5′-CGCCAGTAGACTCCACGAC-3′
PI3K	5′-TCCAAATACCAGCAGGATCA-3′	5′-ATGCTTCGATAGCCGTTCTT-3′
Akt	5′-TACTCATTCCAGACCCACGA-3′	5′-GAGGTTCTCCAGCTTCAGGT-3′
NF-*κ*B	5′-GGAGAAGGCTGGAGAAGATG-3′	5′-GCTCATACGGTTTCCCATTT-3′
IL-6	5′-GGACCAAGACCATCCAATTC-3′	5′-ACCACAGTGAGGAATGTCCA-3′
TNF-*α*	5′-CTAGCCAGGAGGGAGAACAG-3′	5′-GCTTTCTGTGCTCATGGTGT-3′
